# Profile of tuberculosis and its response to anti-TB drugs among tuberculosis patients treated under the TB control programme at Felege-Hiwot Referral Hospital, Ethiopia

**DOI:** 10.1186/s12889-016-3362-9

**Published:** 2016-08-02

**Authors:** Yohannes Zenebe, Yesuf Adem, Daniel Mekonnen, Awoke Derbie, Fetlework Bereded, Minichil Bantie, Begna Tulu, Derese Hailu, Fantahun Biadglegne

**Affiliations:** 1 Department of Medical Microbiology, Immunology and Parasitology, College of Medicine and Health Sciences, Bahir Dar University, Bahir Dar, Ethiopia; 2 Felege Hiwot Referral Hospital, Bahir Dar, Ethiopia; 3 Bahir Dar Regional Health Research Laboratory Institute, Bahir Dar, Ethiopia

**Keywords:** Tuberculosis, Treatment outcomes, Northwest Ethiopia

## Abstract

**Background:**

Tuberculosis (TB) is a global concern for both developing and developed countries. Currently it becomes more complex due to increasing levels of drug resistance and HIV co-infection. Delayed diagnosis and high case load are major factors contributing to continued transmission and failure to the treatment outcome. The study was conducted to determine the profile and treatment outcomes of TB patients at Felege-Hiwot Referral Hospital.

**Methods:**

We analyzed the records of 1761 TB patients registered for treatment in Felege Hiwot Referral Hospital from July 2010 to June 2015. Data on patients’ socio-demographic characteristics, type of TB, HIV status and treatment outcome were analysed. Descriptive statistics and binary logistic regression models were used to present data. The odds ratio and the 95 % confidence intervals were calculated. A *p*-value of < 0.05 was considered statistical significant.

**Results:**

The proportion of smear positive, smear-negative and extra-pulmonary TB were 205 (11.6 %), 548 (31.1 %) and 1008 (57.2 %), respectively. The overall treatment success rate accounts 542(80.8 %) with unsuccessful treatment of 129(19.2 %). The treatment outcome varied by the years from 68.9 to 97.4 %. Among tuberculosis patients, 459(26.1 %) of them were HIV positive. Being HIV positive (AOR = 4.29, 95 % CI, 2.20–8.37 *P* = 0.001), retreatment (AOR = 5.32, 95 % CI, 1.92–14.3, *P* = 0.001), rural residency (AOR = 18.0, 95 % CI, 9.06–37.82, *P* = 0.001) and the age group of 15–24 years (AOR = 2.91, 95%CI, 1.00–8.45, *P* = 0.04) showed statistical significant association for poor treatment outcome.

**Conclusions:**

In the studied region, the overall treatment success rate was still below the WHO target of success rate, 85 %. However, the trend of treatment success rate showed a promising increment. Patients at high risk of unsuccessful treatment outcome should be identified early and given additional follow-up, medical intervention and social support.

## Background

Tuberculosis (TB) is a global concern and recently it becomes more complex due to increasing levels of drug resistance and HIV co-infection [1]. Developing countries, like Ethiopia, have high burden of TB, one of the most serious public health challenges [2]. An increased incidence of TB is found mostly in Africa and Asia, where the highest prevalence of co-infection with HIV is reported [3, 4]. Ethiopia is highly affected by TB and it is ranked 7^th^ among the 22 high TB burden countries by the world health Organization (WHO) [5]. In developing countries, there is high burden of TB and HIV, delayed diagnosis which is a major contributing factor to the continued transmission and failure to the treatment outcome [6]. Patient non-adherence to the anti-TB treatment is also interpreted as a failure of the health care system [7].

Currently, Ethiopia reports treatment success and case detection rates of 83 and 62 % of all forms of TB, respectively (8). The TB control program in Ethiopia introduced the standardized Directly Observed Treatment, Short Course (DOTS) as a pilot programme in 1992 and at Felege Hiwot Referral Hospital in 2000. The standardized DOTS/Stop TB Strategy geographical coverage reached at 100 %. Moreover, at the health facility level, it reached 95 % [8]. However, reports showed that the treatment outcome of TB patients treated under the DOTS program was unsatisfactory [9]. Reports showed that several factors such as male sex, age ≥ 65 years, drug resistance, HIV co-infection, previously treated TB cases and cavitation affect treatment success rate [10–16]. In addition, various social, behavioral, and economic characteristics have been found to be associated with lower treatment success rate [14, 15]. Although the purpose of TB treatment is curing the patient, preventing the spread of TB infection and preventing the emergence of new drug resistant strains, these plans are not achieved in Ethiopia. There is a limited report on the DOTS experiences in Northwest Ethiopia. This study is aimed at evaluating the treatment outcome and associated risk factors for new smear positive pulmonary TB (PTB+), extra pulmonary TB (EPTB) and smear negative pulmonary TB (PTB-) cases registered at Felege-Hiwot Referral Hospital (FHRH) DOTS programme.

## Methods

### Study design, period and population

With a cross sectional retrospective study, all patients diagnosed and treated during the period of July 2010 to June 2015 at FHRH were included in the study to assess TB profile and treatment outcome. The hospital is found in Bahir Dar, the capital city of Amhara National Regional State, which is located 565 km away from Addis Ababa. The current TB diagnosis and treatment strategy in Ethiopia is the stop TB or DOTS strategy. Sputum samples were collected using spot-morning-spot strategy and other clinical samples were collected depending on the site of infection. The collected clinical specimens were examined using the standard Ziehl-Neelsen (ZN) acid fast bacilli (AFB) staining technique, Fluorescent Microscopy (FM) or GeneXpert MTB/RIF assay [8]. Moreover, imaging and pathological techniques might have been used for those PTB- and EPTB cases before being admitted to DOTS programme. For those diagnosed with new active TB, the standard TB treatment regimen such as, 2 months of intensive treatment with Rifampicin, Isoniazid, Pyrazinamide and Ethambutol (2RHZE) followed by 4 months of continuation phase with Rifampicin and Isoniazid (4RH). Likewise for those retreated cases, the intensive phase contains 2 months of streptomycin and the combination of HRZE drugs (2S (HRZE)) and additional 1 month of treatment without streptomycin; 1 (HRZE). Then the continuation phase contains 5 months of treatment with the combination of RH and E; 5 (HR) E [17, 18].

### Human immunodeficiency virus test

The anti-HIV antibody test was used for the screening of HIV/AIDS and it was done according to the manufacturer’s instruction (rapid test currently used in national algorithm for Ethiopia; KHB, Shanghai Kehua Bio-engineering Co., Ltd. China) for screening and positive samples were re-tested with STAT-PACK (Chembio HIV 1/2 STAT-PAK™ Assay, CHEMBIO DIAGNOSTIC SYSTEMS, INC., MEDFORD, NY, USA). Samples giving discordant results in the two tests were re-examined using the tiebreaker (Uni-Gold HIV, Trinity Biotech PLC, Co. Wicklow, Ireland).

### Operational definitions

According to the standard definitions of the National Tuberculosis and Leprosy Control Program guideline (NLCP) adopted from the WHO [19], the following clinical case and treatment outcome definitions were used:

- **Cured:** A patient with bacteriologically confirmed pulmonary TB at the beginning of treatment who was smear or culture-negative in the last month of treatment
- **Treatment completed:** A patient with TB who completed treatment without evidence of failure, but with no record of sputum smear or culture results, in the last month of treatment.
- **Successful treatment outcome:** If PTB patients were cured (i.e., negative smear microscopy at the end of treatment and on at least one previous follow-up test) or completed treatment with resolution of symptoms.
- **Unsuccessful treatment outcome:** If treatment of PTB/EPTB/ patients resulted in treatment failure (i.e., remaining smear-positive after 5 months of treatment), default (i.e., patients who interrupted their treatment for two consecutive months or more after registration), or death.
- **Died:** A TB patient who died from any cause during treatment.
- **Failure:** A TB patient whose sputum smear or culture is positive at month 5 or later during treatment.
- **Lost to follow-up:** A TB patient whose treatment was interrupted for two consecutive months or more.
- **Pulmonary TB, smear-positive:** A patient with at least two sputum specimens which were positive for AFB by microscopy, or a patient with only one sputum specimen which was positive for AFB by microscopy, and chest radiographic abnormalities consistent with active pulmonary TB.
- **Pulmonary TB, smear-negative:** A patient with symptoms suggestive of TB, with at least two sputum specimens which were negative for AFB by microscopy, and with chest radiographic abnormalities consistent with active pulmonary TB (including interstitial or miliary abnormal images), or a patient with two sets of at least two sputum specimens taken at least two weeks apart, and which were negative for AFB by microscopy, and radiographic abnormalities consistent with pulmonary TB and lack of clinical response to one week of broad spectrum antibiotic therapy.
- **Extra pulmonary TB:** This included TB of organs other than the lungs, such as lymph nodes, abdomen, genitourinary tract, skin, joints and bones, meninges, etc. Diagnosis of EPTB was based on fine needle aspiration cytology or biochemical analyses of cerebrospinal/pleural/ascitic fluid or histopathological examination or strong clinical evidence consistent with active EPTB, followed by a decision of a clinician to treat with a full course of anti-tuberculosis chemotherapy. In all the cases of EPTB, sputum examinations and chest radiographs were used to investigate the involvement of lung parenchyma.

### Inclusion and exclusion criteria

All TB patients with complete data like age, sex, treatment outcome were included. Missing of either of these variables were the exclusion criteria.

### Data collection and tools

Demographic and clinical data such as age, sex, type of TB, TB treatment outcome, status of HIV infection, Co-trimoxazole prophylaxis (CPT) and antiretroviral therapy (ART) status were retrieved from TB registry using data extraction sheet.

### Data analysis

All data were entered in to Epi Info 3.1 and analysed using SPSS statistical software package (*IBM Corp. Released 2011. IBM SPSS Statistics for Windows, Version 20.0. Armonk, NY: IBM Corp*.). Descriptive statistics was used to determine differences within the data of variables. All explanatory variables with a *p* value ≤0.2 in the bivariate analysis were included in the multivariate logistic regression model to identify independent predictive variables. Odds ratio (OR) and 95 % confidence intervals (CI) were calculated and the results were considered statistically significant at *p* < 0.05.

## Results

### Socio demographic characteristics of patients

A total of 1761 patients’ document has been reviewed. Of which 1054 (59.9 %) were males. The highest numbers of study participants were urban dwellers, 1142 (64.8 %). The mean age of the study subjects was 28.11 (SD. ±14.25). The proportion of smear positive, smear-negative and extra-pulmonary TB was 205 (11.6 %), 548 (31.1 %) and 1008 (57.2 %), respectively. The age group 15–34 years accounted more than half of the study subjects and the peak age group of 15–24 years presented about one-third of the total TB patients, (Table 1).

**Table 1:** Demographic characteristics and HIV status of Tuberculosis Patients at FHRH, Ethiopia, 2015

Variables	Type of tuberculosis			
	PTB+, N (%)	PTB−, N (%)	EPTB, N (%)	Total, N (%)
Sex				
Female	89 (12.6)	204 (28.9)	414 (58.4)	707 (100)
Male	116 (11.0)	344 (32.6)	594 (56.4)	1054 (100)
Age				
<=14	5 (2.3)	78 (35.6)	136 (62.1)	219 (100)
15–24	102 (16.9)	185 (30.7)	316 (52.4)	603 (100)
25–34	58 (13.1)	126 (28.5)	258 (58.4)	442 (100)
35–44	28 (10.9)	80 (31.0)	150 (58.1)	258 (100)
45–54	4 (3.1)	42 (32.8)	82 (64.1)	128 (100)
≥55	8 (7.2)	37 (33.3)	66 (59.5)	111 (100)
Residence:				
Rural	163 (14.3)	382 (33.5)	597 (52.3)	1142 (100)
Urban	42 (6.8)	166 (26.8)	411 (66.4)	619 (100)
HIV Status:				
Positive	44 (9.6)	164 (35.7)	251 (54.7)	459 (100)
Negative	154 (12.5)	364 (29.5)	717 (58.1)	1235 (100)
Unknown	7 (10.4)	20 (29.9)	40 (59.7)	67 (100)
History of TB				
New	154 (9.8)	497 (31.8)	914 (58.4)	1565(100)
Retreated	13 (40.6)	6 (18.6)	13 (40.6)	32 (100)
Transfer in	36 (23.4)	44 (28.6)	74 (48.1)	154 (100)
Other	2 (20.0)	1 (10.0)	7 (70.0)	10 (100)
Treatment out- come				
Cured	85 (98.8)	0 (0.0)	1 (1.2)	86 (100)
Complete	18 (3.9)	187 (41.0)	251 (55.0)	456 (100)
Died	13 (13.3)	29 (29.6)	56 (57.1)	98 (100)
Failer	1 (100)	0 (0.0)	0 (0.0)	1 (100)
Loss of follow up	3 (10.0)	7 (23.3)	20 (66.7)	30 (100)
Transfer out	85 (7.8)	325 (29.8)	680 (62.4)	1090 (100)
CPT Took				
Yes	33 (11.5)	97 (33.8)	157 (54.7)	287(100)
No	172 (11.7)	451 (30.6)	851 (57.7)	1474(100)

*PTB+*: Smear positive pulmonary tuberculosis, *PTB−*: Smear negative pulmonary tuberculosis, *EPTB*: Extra-pulmonary tuberculosis

### Tuberculosis treatment outcomes

**Fig. 1:**
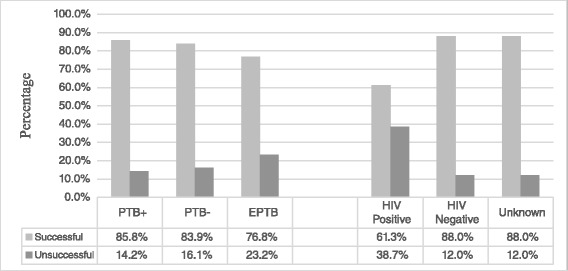
The distribution of tuberculosis treatment outcome based on of types of TB and HIV status among TB patients at FHRH, 2015

Of the total TB patients, 1090 (61.9 %) were transferred out to other health facilities. Thus, their treatment outcome status were not known and excluded from the treatment outcome analysis. Of the total TB patients with known treatment outcome, the treatment success rate was 542(80.8 %) with unsuccessful treatment of 129(19.2 %). The unsuccessful treatment outcomes shared to 30(4.5 %) defaulted, 98(14.6 %) died and 1(0.001 %) failed. Among all TB patients, 459(26.1 %) of them were sero-positive for HIV/AIDS (Table 1). The majority of TB patients who have unsuccessful treatment outcome were among HIV positive patients, 70 (38.7 %) (Fig. 1). The death rate of PTB+, PTB- and EPTB were 13(13.3 %), 29(29.6 %) and 56(57.1 %), respectively (Table 1).

### Trend of tuberculosis treatment outcomes

**Fig. 2:**
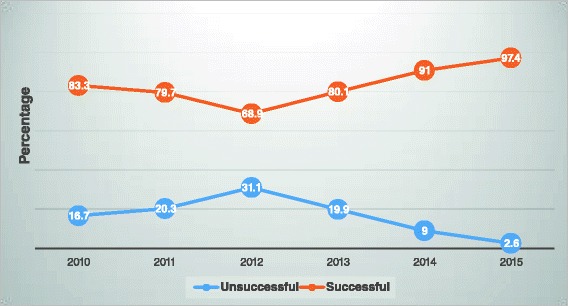
The 5 years trend of TB treatment outcome rate among TB patients at FHRH, 2015

Over a period of 5 years, the treatment outcomes under DOTS program of TB patients varied from years to years. The successful treatment outcomes from 2010 to 2012 were decreased from 83.3 to 68.9 %. But, from 2013 onwards it showed an increase of successful treatment outcomes from 80.1 to 97.4 %. The detail information is described in Fig. 2.

### Factors associated with TB treatment success rate

In the multivariate logistic regression model, HIV positive TB patients were about 4 times more likely to have unsuccessful TB treatment than whose sero-status was unknown (AOR = 4.29, 95 % CI, 2.20–8.37 *P* = 0.001), while a decreased treatment success rate was observed with the patients’ age group 15–24 years (AOR = 2.91, 95 % CI, 1.00–8.45, *P* = 0.04). Furthermore, a lower treatment success rate was documented among retreated TB patients (AOR = 5.32, 95 % CI, 1.92–14.3, *P* = 0.001). On the other hand, patients from urban areas had a significantly higher treatment success rate compared to cases from rural locations (AOR = 18.0, 95 % CI, 9.06–37.82, *P* = 0.001) (Table 2).

**Table 2:** Association of poor treatment outcome with possible predictors among tuberculosis patients at FHRH, 2015

Predictor variables	TB treatment outcomes		COR (95 % CI), *P*-value	AOR (95 % CI), *P*-value
	Unsuccessful N (%)	Success full N (%)		
Sex				
Female	63 (24.4)	195 (75.6)	0.58 (0.4–0.87), 0.007	0.26 (0.06–1.13),0.26
Male	66 (16.0)	347 (84.0)	1	1
Age				
<=14	6 (12.5)	42 (87.5)	2.57 (0.76–8.71),0.13	2.4 (0.63–9.12),0.20
15–24	24 (8.3)	266 (91.7)	4.08 (1.56–10.68), 0.04	2.91 (1.00–8.45),0.04
25–34	47 (27.2)	126 (72.8)	0.98 (0.39–2.50), 0.98	1.22 (0.43–3.46),0.70
35–44	30 (31.9)	64 (68.1)	0.78 (0.29–2.07),0.62	1.10 (0.37–3.29),0.86
45–54	15 (37.5)	25 (62.5)	0.61 (0.20–1.80), 0.37	1.34 (0.39–4.84),0.61
≥55	7 (26.9)	19 (73.1)	1	1
Residence				
Rural	42 (72.4)	16 (27.6)	15.87 (8.55–29.47), 0.00	18.0 (9.06–37.82),0.001
Urban	87 (14.2)	526 (85.8)	1	1
Types of TB				
PTB+	17 (14.2)	103 (85.8)	1.83 (1.03–3.24),0.03	1.90 (0.92–3.90),0.08
PTB-	36 (16.1)	187 (83.9)	1.57 (1.01–2.43),0.04	1.46 (0.86–2.46),0.154
EPTB	76 (23.2)	252 (76.8)	1	1
HIV Status				
Positive	70 (38.7)	111 (61.3)	4.6 (3.06–6.93),0.001	4.29 (2.20–8.37), 0.001
Negative	56 (12.0)	412 (88.0)	4.62 (1.33–16.02),0.016	3.70 (0.88–16.17),0.07
Unknown	3 (13.6)	19 (86.4)	1	1
History of TB				
New	117 (21.9)	418 (78.1)	1	1
Retreated	7 (36.8)	12 (63.2)	6.27 (2.50–15.71),0.001	5.32 (1.92–14.3),0.001
Transfer in	5 (4.5)	107 (95.5)	10.48 (0.18–1.24),0.13	0.23 (0.08–0.70),0.01
Other	0 (0)	5 (100)		
CPT Took				
Yes	42 (34.4)	80 (65.6)	2.79 (1.80–4.32),0.001	1.32 (0.65–2.61),0.45
No	87 (15.8)	462 (84.2)	1	1

*PTB+*: Smear positive pulmonary tuberculosis, *PTB−*: Smear negative pulmonary tuberculosis, *EPTB*: Extra-pulmonary tuberculosis, *AOR*: adjusted odds ratio, *COR *: cruds odds ratio

## Discussion

In our study with the microscopically confirmed TB cases in FHRH DOTS clinic, we observed a successful outcome of TB treatment in 80.8 % of the patients, which is less than the target level set by the WHO for successful outcomes of 85 %. However, it is comparable to the national report of the successful outcome of TB treatment in Ethiopia (83 %) [8]. Moreover the treatment success rate among the 22-high TB burden countries varied from 60 to 93 %, with an average of 83 % [4, 8, 20]. On the other hand, our finding is still below other findings reported in Tigray, Ethiopia, 89.2 % [21] and in Spain, 89 % [22]. But the treatment success in our study was better than the studies conducted in Southern Ethiopia, 49 % [23], and Finland, 70.1 % [24].

The treatment success rate was poor in 2010–2012, although a prominent improvement was observed in 2013–2015. This improvement could be due to an improved access to TB control services, particularly community-based interventions, the expansion of TB treatment centers and a regimen change of the continuation phase to RH, which lasts 4 months. This finding is supported by the study conducted at the Southern Ethiopia [25].

The proportion of PTB^+^, PTB^−^ and EPTB were 205 (11.6 %), 548 (31.1 %) and 1008 (57.2 %), respectively. This is in line with other studies reported elsewhere in the country [9, 21, 26, 27]. However, the vice versa result has been declared from Gambella region [28]. The high level of EPTB in this study might be due to the over diagnosis of EPTB using clinical data, imaging and pathologic evidence. Moreover, the large number of PTB^−^ and EPTB cases might be due to the high proportion of TB - HIV co-infection which is quite common among these patients in a high TB and HIV burden setting [29].

Unsuccessful treatment outcome was more frequent (AOR = 5.32, 95 % CI, 1.92–14.3, *P* = 0.001) among retreated cases than among those newly treated. This was similar to the study conducted in Tigray region, Ethiopia [21]. Among TB patients, the majority of them were EPTB cases and the most unsuccessful outcome of TB treatment at (23.2 %) was observed in these patients.

In this study, the proportion of TB/HIV co-infection was found to be 459 (26.0 %). The majority of HIV positive patients (29.9 %) were among PTB- patients, indicating that HIV testing before treatment is crucial. This finding was higher than the study conducted in India [30]. However, previous studies conducted at Gondar University Hospital showed that high proportions (52.5 %) of TB patients were co-infected with HIV. [31, 32]. Moreover, multivariate logistic regression analysis showed that, being HIV positive was one of the independently associated risk factor for poor treatment outcome (AOR = 4.29, 95 % CI, 2.20–8.37, *P* = 0.001). This finding was in agreement to other study in Zimbabwe [33]. This might be due to the low level of immunity and drug mal-absorption among HIV patients. This is supported by other studies [27, 34, 35]. Our results indicate the necessity of strengthening interventions to reduce TB-HIV co-infection in the study region.

It is well studied that retreated patients are more vulnerable for the development of MDR-TB and poor treatment outcome [36]. In our finding, unsuccessful treatment outcome was higher among retreated TB cases than new TB patients (AOR = 5.32, 95 % CI, 1.92–14.3, *P* = 0.001). Similar finding was declared from the study conducted in South India [37]. Rural residence was also significantly associated with the poor treatment outcome of TB patients (AOR = 18.0, 95 % CI, 9.06–37.82, *P* = 0.001). The same finding was reported by Hailu MD et al. [25]. This might be due to awareness differences between rural and urban populations. Tuberculosis patients in the age group 15–24 years were more likely for the unsuccessful treatment outcome than other age group (AOR = 2.91, 95 % CI, 1.00–8.45, *P* = 0.04). However, Berhe G et al. in 2012 [21] and Tessema et al. in 2009 [9] reported different pattern. This finding is unusual and it is recommended to be more studied. In this study, there was no difference in success rates by sex. This was also supported by other studies conducted elsewhere in Ethiopia [21, 38].

In this study, we observed a default rate at 4.5 %, which was found to be lower compared to other studies in Ethiopia, 18 % [9], Switzerland, 16 % [39], Germany, 10 % [40], and Sweden, 7 % [41]. In Ethiopia a standardized TB prevention and control programme is incorporating DOTS. This might be one of the reasons for the smaller proportions of default rate in this study. However, the presence of default is a major public health problem that the patients may remain infectious and even develop MDR-TB.

## Conclusions

In conclusion, the overall treatment success rate in the current study (80.8 %) was still below the WHO target of success rate (85 %). However, the prominent improvement has been observed in 2013–2015. In the studied region, HIV-TB co-infection, young age (15–24 years), rural residence and retreatment of patients were found to be predictors for the poor treatment outcome. Based on our finding, we recommend that patients at high risk of unsuccessful treatment outcome should be identified early and given additional follow-up, medical intervention and social support.
